# Spontaneous onset and transplant models of the Vk*MYC mouse show immunological sequelae comparable to human multiple myeloma

**DOI:** 10.1186/s12967-016-0994-6

**Published:** 2016-09-06

**Authors:** Rachel E. Cooke, Nicholas A. Gherardin, Simon J. Harrison, Hang Quach, Dale I. Godfrey, Miles Prince, Rachel Koldej, David S. Ritchie

**Affiliations:** 1 ACRF Translational Research Laboratory, Royal Melbourne Hospital, Parkville, VIC 3010 Australia; 2 Cancer Immunology Program, Peter MacCallum Cancer Centre, University of Melbourne, East Melbourne, VIC 3002 Australia; 3 Department of Medicine, University of Melbourne, Melbourne, Australia; 4 Department of Microbiology and Immunology, Peter Doherty Institute for Infection and Immunity, University of Melbourne, Melbourne, VIC 3000 Australia; 5 Australian Research Council Centre of Excellence in Advanced Molecular Imaging, University of Melbourne, Parkville, 3010 Australia

**Keywords:** Myeloma, Vk*MYC, Immunology, Microenvironment, IFNγ, CD8, TEM

## Abstract

**Background:**

The Vk*MYC transgenic and transplant mouse models of multiple myeloma (MM) are well established as a research tool for anti-myeloma drug discovery. However, little is known of the immune response in these models. Understanding the immunological relevance of these models is of increasing importance as immunotherapeutic drugs are developed against MM.

**Methods:**

We set out to examine how cellular immunity is affected in Vk*MYC mouse models and compare that to the immunology of patients with newly diagnosed and relapsed/refractory MM.

**Results:**

We found that there were significant immunological responses in mice developing either spontaneous (transgenic) or transplanted MM as a consequence of the degree of tumor burden. Particularly striking were the profound B cell lymphopenia and the expansion of CD8^+^ effector memory T cells within the lymphocyte population that progressively developed with advancing disease burden, mirroring changes seen in human MM. High disease burden was also associated with increased inflammatory cytokine production by T lymphocytes, which is more fitting with relapsed/refractory MM in humans.

**Conclusions:**

These findings have important implications for the application of this mouse model in the development of MM immunotherapies.

*Trial registration* LitVacc ANZCTR trial ID ACTRN12613000344796; RevLite ANZCTR trial ID NCT00482261

## Background

Multiple myeloma (MM) is a malignancy of plasma cells that reside within a supportive niche in the bone marrow (BM) [1, 2]. The majority, if not all, patients have a pre-malignant phase of monoclonal gammopathy of undetermined significance (MGUS) [3, 4]. With evolution of disease, patients variably develop organ damage that can result in lytic bone lesions, hypercalcaemia, renal failure and anaemia [1, 2]. Acquired immune paresis complicates advanced disease due to residual hypogammaglobulinaemia, B cell hypoplasia [5, 6], the effects of cumulative chemotherapies [7–9] and an ageing and exhausted T cell population [10, 11]. Although MM is largely incurable, the advent of immunomodulatory agents and proteasome inhibitors has significantly increased the life expectancy of patients with this disease [2, 12].

Numerous factors in MM are involved in the velocity of disease progression and they are acquired in a non-stochastic but cumulative fashion over time [13]. These include immune-editing and oncogenic mutations, which both promote evasion from immune surveillance and resistance to apoptosis. By utilizing knowledge of driver mutations, mouse models of disease have been developed. These mouse models are useful tools to study anti-myeloma therapies; however, a deeper appreciation of their relevance to clinical disease is required. It is understood that, unlike human MM, the mice used for these models are genetically uniform and the tumours often have a low mutational burden [14]. Less is known about the immunological similarities and differences between human disease and the Vk*MYC mouse models.

The Vk*MYC mouse model has been established as an investigative tool for anti-myeloma drug therapy [15, 16]. The disease mechanism involves spontaneous AID-dependent activation of MYC in post germinal B cells; an event that is not required for MM development in humans, although complex c-myc gene rearrangements and translocations often occur with disease progression [17]. It has been argued that there is no true “MGUS” period in the Vk*MYC model, rather the slow accumulation of malignant cells [18]. Others have delineated a transition from “smoldering” to active myeloma period based on differences in BM vascularization [19].

It is also possible to induce a plasma cell malignancy in congenic C57BL/6 mice by transplanting cryopreserved cells from the spleen of a diseased transgenic Vk*MYC mouse [16]. The advantage to this method is the greatly reduced time to disease state (weeks as opposed to 12–18 months with the transgenic model). However, the injected malignant cells are potentially quite different from their original BM resident counterparts, in that the transplanted model more readily develops extramedullary disease.

Research using these models has been mainly concentrated on the development of new tumouricidal agents [16]; however, the role of the host immune response to malignancy is increasingly being realized, particularly in the context of immune therapies such as immunomodulatory drugs and checkpoint blockade inhibitors [20–22]. When assessing the role of novel immunomodulatory drugs in mouse models, it is important to understand their effects in context of the immune microenvironment.

We investigated the immunological relevance of different models of myeloma utilizing the Vk*MYC mouse and compared those findings to our analysis of T cells in human MM. In order to establish whether T cell aberrancies that are seen in relapsed/refractory disease are present in newly diagnosed MM, we analysed peripheral blood mononuclear cells (PBMCs) from patients with newly diagnosed MM (NDMM) and relapsed/refractory MM (RRMM), and compared with healthy blood donors from Australian Red Cross Blood Service (ARCBS). To evaluate whether the transgenic or transplant versions of the Vk*MYC mouse model would replicate our clinical findings, we examined a cohort of aged transgenic Vk*MYC mice with spontaneous onset tumours and two of the more commonly used transplant clones in our laboratory: Vk#4929 [16] and Vk#31.

We found that the immunological consequences of disease in the Vk*MYC mouse models were similar whether spontaneous onset (transgenic) or transplant models were used, providing that macroscopically evident extramedullary disease was not present. Advanced disease in these models replicated T cell fate most similar to that observed in RRMM; however, there may be a “window” with the transgenic Vk*MYC model that is comparable to new-onset disease in humans.

## Methods

### Human samples and cells

All research was conducted in full conformance with principles of the “Declaration of Helsinki”, Good Clinical Practice and within the laws and regulations of Australia. Cryopreserved peripheral blood mononuclear cells (PBMCs) and bone marrow mononuclear cells (BMMCs) were analysed from MM patients enrolled in two clinical trials at baseline: LitVacc (ANZCTR trial ID ACTRN12613000344796, which recruited newly diagnosed, untreated patients), and RevLite (ANZCTR trial ID NCT00482261, which recruited relapsed/refractory patients). As a control group, PBMCs from ARCBS donors were used.

### Mouse samples and cells

C57BL/6 mice were bred and housed at the Peter MacCallum Cancer Center (PMCC) animal house. The studies were performed in accordance to PMCC Animal Ethics Committee approved procedures.

#### Transgenic Vk*MYC

BM and spleen were obtained from 15 transgenic Vk*MYC mice with six age-matched controls in two consecutive culls (14–18 months of age).

#### Transplant Vk*MYC

Vk#4929 and Vk#31 plasma cell clones were obtained from transgenic Vk*MYC mouse spleens and cryopreserved. These clones were chosen based on experience of previous transplants where mice had a median survival of 8 and 14 weeks respectively. Recipient C57BL/6 mice were injected via tail vein with approximately 1–2 × 10^5^ cells. Mice were sacrificed once a notable monoclonal band (M-band) was present on serum protein electrophoresis and/or significant morbidity, which occurred at 12–16 weeks post-transplant (Vk#4929) and 11–19 weeks post-transplant (Vk#31). The results shown are representative data from two independent transplant cohorts for Vk#4929 and three consecutive culls from one transplant cohort for Vk#31, with six age-matched controls.

#### Cell preparation

Cell suspensions for flow cytometry were prepared by extracting BM from dissected bones, and the spleens were bluntly crushed in T cell media* before passing through a 40 μm filter. Cells were resuspended in T-cell media and aliquots of 5 × 10^6^–1 × 10^7^ cells were stained.

#### *T-cell media

RPMI-1640 (Gibco) with 10 % foetal bovine serum, β-mercaptoethanol, non-essential amino acids, HEPES, Na-pyruvate, glutamax and penicillin/streptomycin.

### Flow cytometry

#### Flow cytometry was performed using directly conjugated antibodies

*Anti*-*human* CD3ε (UCHT1, BD Biosciences), CD4 (OKT3, Biolegend), CD8α (SK1, Biolegend), CD14 (M5E2, Biolegend), CD19 (HIB19, Biolegend), CD27 (0323, Biolegend), CD33 (IV M-505, Biolegend), CD45RA (HI100, Biolegend), CD57 (NK-1, BD Biosciences), pan-TCRγδ (11F2, BD Biosciences), IFNγ (4S.B3, Biolegend) and IL17a (eBio64DEC17, eBioscience).

*Anti*-*mouse* TCRβ (H57-597, eBioscience), CD4 (GK1.5, Biolegend), CD8α (53-6.7, eBioscience), CD19 (1D3, BD Pharmingen), B220 (RA3-6B2, eBioscience), CD44 (IM7, BD Pharmingen), CD62L (MEL-14, BD Pharmingen), CD138 (281-2, Biolegend), IFNγ (XMG1.2, eBioscience) and IL-17A (TC11-18H10.1, Biolegend).

Fluorescence-minus-one (FMO), internal negative and/or isotype controls were included to assist with gating.

*Human* Dead and irrelevant cells were excluded using Zombie Yellow viability dye (Biolegend) and CD14, CD19 and CD33. Lymphocytes were then gated using forward and side scatter followed by doublet removal. *Intracellular cytokine staining* PBMC/BMMC were stimulated for 4 h with PMA and ionomycin in the presence of monensin. Cells were harvested, surface stained and then permeabilised with Fix/Perm reagents (BD Bioscience) as per manufacturer’s instructions, prior to intracellular staining.

*Mouse* Lymphocytes were gated using forward and side scatter, followed by doublet and dead cell removal using Fixable Yellow (Invitrogen). *Intracellular cytokine staining* BMMC were stimulated for 4 h with 2 μl/ml Cell Stimulation Cocktail Plus Protein Transport Inhibitors (eBioscience). Cells were harvested, surface stained and then permeabilised with Fix/Perm (BD Bioscience) as per manufacturer’s instructions, prior to intracellular staining.

Analysis was performed on BD LSR or BD LSR Fortessa cell analysers, and interpreted using FlowJo software (Treestar).

### Cytometric bead array (CBA) assay

BM was extracted and suspended in 5 ml T cell media. After centrifugation, the supernatant was collected and stored at −20 °C. Cytokine analysis was performed using the BD mouse Th1/Th2/Th17 CBA kit as per manufacturer’s instructions.

### Serum protein electrophoresis

Mouse serum was obtained by retro-orbital blood sampling and serum gel electrophoresis was performed using the Hydasys 2 Scan (Sebia). An independent pathologist analysed the results.

### Histopathology

BM trephines were prepared from dissected sternum from culled mice. These were fixed for 24 h in 4 % paraformaldehyde, and decalcified in 10 % EDTA for 10–14 days. Paraffin embedded BM trephines were prepared by the Petermac Microscopy and Histology Core Facility. CD138 immunohistochemistry was performed using rat anti-mouse CD138 antibody and biotin goat anti-rat immunoglobulin (BD Pharmingen) with streptavidin-HRP and DAB staining.

### Statistical analysis

Data was analysed using GraphPad Prism software. Statistical significance was determined using the Wilcoxon signed rank test (paired samples) or the Mann–Whitney test (unpaired samples) to compare two groups, or the student’s t test with the Holm-Sidak method applied for multiple comparisons. Pearsons correlation was used to calculate r^2^ values. *p < 0.05, **p < 0.01, ***p < 0.001.

## Results

### Transgenic and transplant Vk*MYC mouse models show differing disease dynamics, but consistently develop B lymphopenia with advanced disease

MM patients requiring treatment for their disease have organ damage in the form of anaemia, renal failure and/or lytic bone lesions, which has been shown to be replicated in the Vk*MYC mouse models [16]. MM bone marrow infiltrates can be variable and do not necessarily directly correlate with end-organ damage; however, a BM plasma cell infiltrate of >50 % is considered high risk [23]. Serum protein electrophoresis, to identify paraprotein produced by the malignant cells, is often used as a surrogate marker for BM disease progression or response to treatment. Although human MM consists of heterogeneous clones [24], monoclonal banding is usual but one or more additional clones can develop in some with disease progression.

**Fig. 1:**
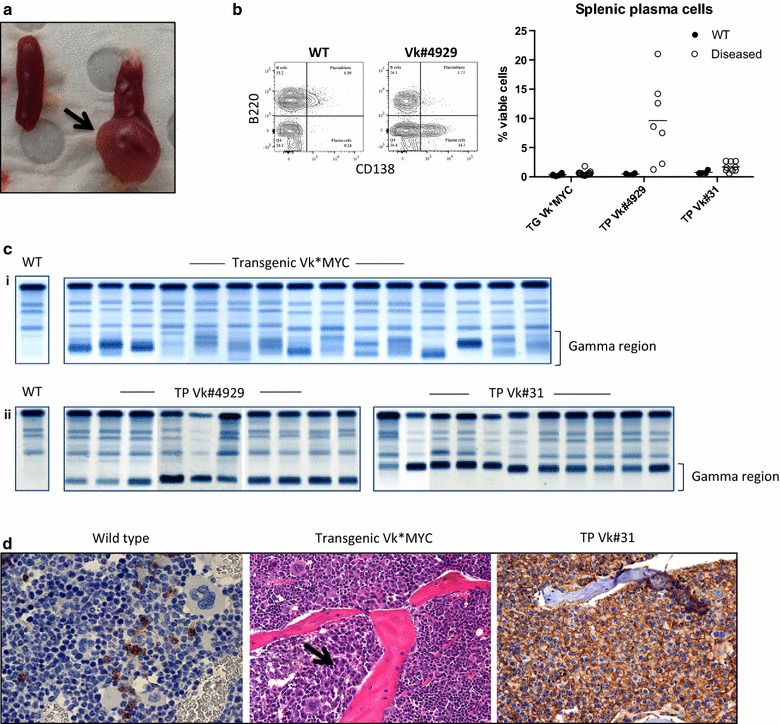
Disease characteristics of the transgenic and transplant Vk*MYC models. **a** Dissected spleen (*left*) wild type control (*right*) diseased Vk#4929 with a large plasmacytoma (*arrow*). **b** Plasma cells in the spleen of wild type and diseased Vk*MYC mice. Representative FACS plot is shown to the *left*: plasma cells are defined as CD138^+^B220^−^ events and expressed as % viable cells. **c** Serum protein electrophoresis in individual mice at time of cull: (*i*) Transgenic Vk*MYC showing predominantly oligoclonal banding in the gamma region. (*ii*) Transplant Vk#4929 and Vk#31 showing monoclonal banding in the fast or slow gamma regions respectively. **d** BM trephine immunohistochemistry. *Left* CD138 staining showing normal numbers of plasma cells in wild type control. *Middle* H&E showing patchy plasma cell infiltrate in transgenic Vk*MYC (*arrow*). *Right* CD138 staining showing a diffuse infiltrate of plasma cells in transplant Vk#31

In order to establish comparisons with the human data, we elected to cull mice at time points when they were likely to have developed a significant burden of disease within the BM (described in “Methods” section). As anticipated, transgenic Vk*MYC mice had slow onset, relatively benign disease without major manifestations of end organ damage at the time of cull, whereas the transplanted mice characteristically exhibited rapidly progressive disease and clinical signs of distress. Mice transplanted with Vk#4929 variably developed bulky splenomegaly, sometimes with large plasmacytomas (Fig. 1a), or hind limb weakness. Flow cytometry confirmed extramedullary disease with an excess of plasma cells in the spleen (Fig. 1b).

**Fig. 2:**
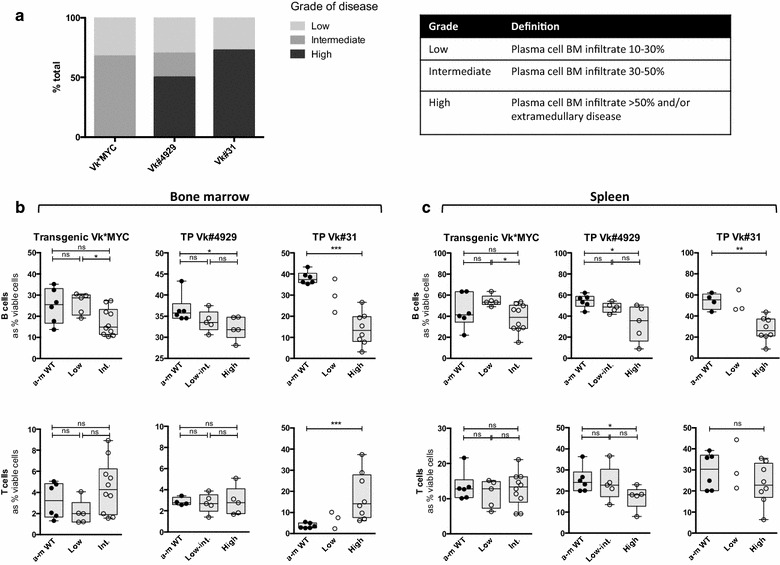
Disease characteristics of the transgenic and transplant Vk*MYC models. **a** *Bar graph* depicting grade of disease in the Vk*MYC mouse models. **b** BMMC lymphocyte analysis in Vk*MYC mouse models: Box and whisker plots showing B lymphocyte (*top*) and T lymphocyte (*bottom*) populations. B cells are identified as B220^+^CD19^+^ events and T cells are identified as TCRβ^+^B220^−^ events, and are expressed as % viable cells from the FS/SS lymphocyte gate. (*Left*) Transgenic Vk*MYC (*middle*) Transplant Vk#4929 (*right*) Transplant Vk#31. *Closed circles* represent age-matched wild type control (a-m WT), *open circles* represent diseased mice [which are subdivided into low, intermediate (int.) or high-grade disease as described in **a**]. **c** Splenic lymphocyte analysis in Vk*MYC mouse models: Box and whisker plots (as described for **b**)

Transgenic Vk*MYC mice developed oligoclonal banding but, in some cases, one or two clones became dominant over time forming monoclonal or biclonal bands (Fig. 1c). In contrast, the transplant clones (Vk#4929 and Vk#31) were picked because of their monoclonality, and a replicative monoclonal band was seen in all mice transplanted. Bone marrow histology also differed, with transgenic mice tending to have patchy BM infiltrates (Fig. 1d, *middle*) as compared to transplanted mice that more often had diffuse BM infiltrates (Fig. 1d, *right*).

We used this information to divide the mice into groups based on grade of disease: low grade disease was defined as a BM plasma cell infiltrate of 10–30 %, intermediate disease 30–50 % and high grade disease ≥50 % plasma cell infiltrate (and/or the presence of extramedullary disease). In keeping with the clinical features, transgenic mice had low-intermediate grade disease but the transplanted mice usually had high-grade disease (Fig. 2a).

In both BM and spleen there was a progressive reduction in the proportion of B cells in all Vk*MYC subtypes with increasing tumour burden, that reached significance with high-grade disease (Fig. 2b, c). This is consistent with data shown in human clinical cohorts of MM, where a profound B lymphopenia has been described in both newly diagnosed and relapsed MM [5, 6]. There was a significant increase in the proportion of T cells in transplant Vk#31 with high-grade disease in the BM, but not the spleen, suggesting that this may not purely be a reciprocal change due to B lymphopenia (Fig. 2b, c *right*). On the other hand, there was a simultaneous reduction in the proportion of T cells in the spleen of transplant Vk#4929 with high-grade disease, reflecting the splenic plasma cell infiltrate seen in this model (Fig. 2b, c *middle*).

### Comparison of CD4^+^ and CD8^+^ T lymphocyte populations in human MM and the Vk*MYC mouse models

Our group has previously shown that patients with RRMM have marked CD4^+^ T lymphopenia that does not recover with lenalidomide/dexamethasone therapy [25]. To determine whether this is also true in newly diagnosed MM, we analysed PBMCs from NDMM and RRMM patients, and compared with healthy donors.

**Fig. 3:**
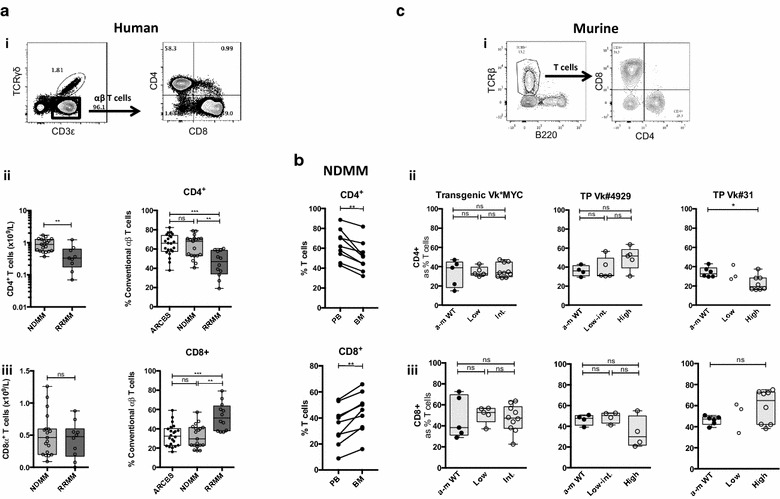
CD4^+^/CD8^+^ T lymphocyte analysis in human MM and Vk*MYC mouse models. **a** Human PBMC CD4^+^/CD8^+^ T lymphocyte analysis. (*i*) FACS plots showing representative staining: CD4^+^/CD8^+^ T cells are expressed as % αβ T cells (CD3ε^+^ TCRγδ^−^ events). (*ii*–*iii*) Box and whisker plots showing (*ii*) CD4^+^ T cells and (*iii*) CD8^+^ T cells as an absolute number (*left*) and as a proportion of αβ T cells (*right*). *Clear boxes* ARCBS donors, *light grey boxes* NDMM, *dark grey boxes* RRMM. **b** Human T lymphocyte matched pair analysis of PBMC and BMMC samples in NDMM (as described for **a**). **c** Murine BMMC CD4^+^/CD8^+^ T lymphocyte analysis in Vk*MYC mouse models. (*i*) FACS plots showing representative staining: CD4^+^/CD8^+^ T cells are expressed as % T cells (TCRβ^+^B220^−^ events). (*ii*–*iii*) Box and whisker plots showing (*ii*) CD4^+^ T cells (*iii*) CD8^+^ T cells. (*Left*) Transgenic Vk*MYC (*middle*) Transplant Vk#4929, (*right*) Transplant Vk#31. *Closed circles* represent a-m WT, *open circles* represent diseased mice [which are subdivided into low, intermediate (int.) or high-grade disease as described in Fig. 2a]

We show that there was a decrease in both the absolute number and proportion of CD4^+^ T lymphocytes in RRMM (accompanied by a reciprocal increase in CD8^+^ T lymphocytes) that was not present in NDMM (Fig. 3a). NDMM patients had a comparable CD4^+^/CD8^+^ T cell profile to that of healthy donors (Fig. 3a *right*), which were derived from blood donors with normal white cell counts. As expected, BM samples from NDMM patients showed a decreased CD4:8 ratio compared with PB (Fig. 3b).

BM T lymphocyte populations appeared unaltered in transgenic Vk*MYC and transplant Vk#4929, but there were notable changes occurring in transplant Vk#31 with high-grade disease (Figs. 2b, 3c). As discussed previously, the increase in % T lymphocytes in Vk#31 with high grade disease could represent a reciprocal change to the B lymphopenia occurring (Fig. 2b); however, the change in CD4:CD8 ratio with high-grade disease suggested a preferential expansion of CD8^+^ T cells and/or loss of CD4^+^ T cells (Fig. 3c).

### Advanced disease is associated with increased inflammatory cytokine production

**Fig. 4:**
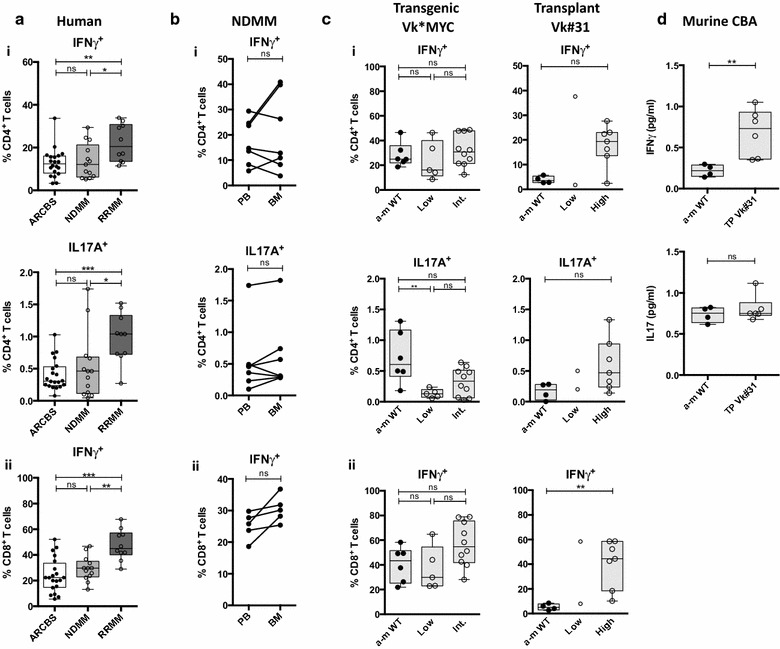
T lymphocyte intracellular cytokine staining in human MM and Vk*MYC mouse models. **a** Human PBMC CD4^+^/CD8^+^ T lymphocyte intracellular cytokine staining (ICS). Box and whisker plots showing (*i*) % IFNγ^+^ (*top*) and % IL-17A^+^ (*bottom*) expressing CD4^+^ T lymphocytes (*ii*) % IFNγ^+^ expressing CD8^+^ T cells. *Clear boxes* ARCBS donors, *light grey boxes* NDMM, *dark grey boxes* RRMM. **b** ICS matched pair analysis in PBMC and BMMC samples in NDMM (as described for **a**). (*i*) % IFNγ^+^ (*top*) and % IL-17A^+^ (*bottom*) expressing CD4^+^ T lymphocytes (*ii*) % IFNγ^+^ expressing CD8^+^ T cells. **c** Murine BMMC CD4^+^/CD8^+^ T lymphocyte ICS in Vk*MYC mouse models. Box and whisker plots showing (*i*) % IFNγ^+^ (*top*) and % IL17^+^ T cells (*bottom*) expressing CD4^+^ T lymphocytes (*ii*) % IFNγ^+^ expressing CD8^+^ T cells. (*Left*) Transgenic Vk*MYC, (*Right*) Transplant Vk#31. *Closed circles* represent a-m WT, open circles represent diseased mice (which are subdivided into low, intermediate (int.) or high-grade disease as described in Fig. 2a). **d** Box and whisker plots showing IFNγ (*top*) and IL17 (*bottom*) concentration in BM supernatants from transplant Vk#31 and age-matched WT controls by cytometric bead array (CBA) analysis

Th17 cells have been shown to be increased in the PB and BM microenvironment of patients with MM compared to normal, as well as elevated levels of IL-17, Th17-polarising cytokines [26–28] and IFNγ [28] in the BM. We found significantly increased IFNγ-producing CD4^+^ and CD8^+^ T lymphocytes and (to a much lesser degree) increased IL-17A-producing CD4^+^ T lymphocytes in PBMC samples from patients with RRMM compared to levels seen in NDMM or healthy controls (Fig. 4a). The proportion of cytokine-producing T lymphocytes was comparable between PBMC and BMMC from NDMM patient samples (Fig. 4b).

In Vk#31, but not transgenic Vk*MYC mice, there was a trend towards increased IFNγ- and IL17A-producing CD4^+^ T lymphocytes in high-grade disease (Fig. 4c(i)). IFNγ-producing CD8^+^ T lymphocytes increased with disease burden in both mouse models, but were only significantly higher in Vk#31 with high-grade disease (Fig. 4c(ii)). Increased IFNγ was also evident in BM supernatants from Vk#31 as compared with controls; no significant difference was noted in IL17A levels (Fig. 4d). Increased inflammatory cytokine production was not seen in transplant Vk#4929 with high-grade disease (data not shown).

### Shifts within memory T cell subsets, evident from first diagnosis in human MM, are replicated in the transgenic Vk*MYC mouse model

Shifts within T cell subset populations have been described in ageing populations [10, 29, 30] and in patients with chronic inflammation or malignancy [31]. In both cases, naïve T cells are depleted with reciprocal accumulation of variably described “antigen-experienced cells” (namely effector memory T (T_EM_) cells or terminally differentiated effector memory T (T_EMRA_) cells) that share characteristics of an exhausted phenotype and low proliferative capacity.

**Fig. 5:**
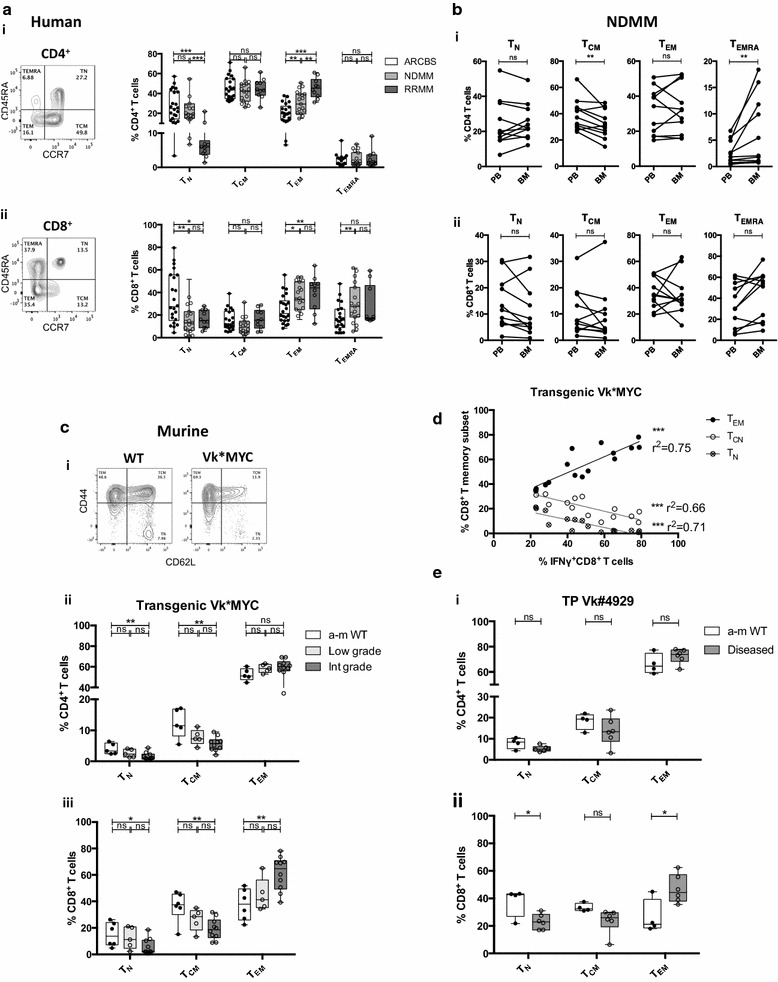
T cell memory subset analysis in human MM and Vk*MYC mouse models. **a** Human PBMC CD4^+^/CD8^+^ T cell memory subset analysis. (*i*–*ii*) Box and whisker plots showing T cell memory subsets (*i*) CD4^+^ T cells (*ii*) CD8^+^ T cells. FACS plots showing representative staining are shown to the *left*: T cell subsets are expressed as %CD3ε^+^CD4^+^ or %CD3ε^+^CD8^+^ T cells and are defined as naïve/T_N_ (CD45RA^+^CCR7^+^), central memory/T_CM_ (CD45RA^−^CCR7^+^), effector memory/T_EM_ (CD45RA^−^CCR7^−^), and terminally differentiated effector memory T cells/T_EMRA_ (CD45RA^+^CCR7^−^). *Clear boxes* ARCBS donors, *light grey boxes* NDMM, *dark grey boxes* RRMM. **b** T cell memory subset matched pair analysis in PBMC and BMMC from NDMM patients (as described in **a**). (*i*) CD4^+^ T cells (*ii*) CD8^+^ T cells. **c** Murine BMMC T cell memory subset analysis in transgenic Vk*MYC. (*i*) FACS plots showing representative staining: T cell subsets are expressed as %TCRβ^+^CD8^+^ or %TCRβ^+^CD8^−^ (CD4^+^) and are defined as naïve/T_N_ (CD44^−^CD62L^+^), central memory/T_CM_ (CD44^+^CD62L^−^) and effector memory/T_EM_ (CD44^+^CD62L^−^). (*ii*–*iii*) Box and whisker plots comparing WT to diseased transgenic Vk*MYC. (*ii*) CD4^+^ T cells (*iii*) CD8^+^ T cells. *Closed circles* represent a-m WT, *open circles* represent diseased mice (which are subdivided into low, intermediate (int.) or high-grade disease as described in Fig. 2a). **d** Dot plot showing correlation between %CD8^+^ T_EM_ and %IFNγ^+^CD8^+^ T cells in transgenic Vk*MYC mice. **e** Murine BMMC T cell memory subset analysis in a diseased cohort of transplant Vk#4929 (flow cytometry as described for **c**). *Clear boxes*/*closed circles* represent a-m WT, *dark grey boxes*/*open circles* represent diseased mice

To investigate whether similar changes in T cell profile occurred in MM patients, we next examined T cell memory subsets, using CCR7 and CD45RA to describe naïve (T_N_), central memory (T_CN_), effector memory (T_EM_) and terminally differentiated effector memory (T_EMRA_) T cell memory subsets. We showed that in RRMM patients there were significant changes in the memory T cell profile, with a marked reduction in the proportion of CD4^+^ and CD8^+^ T_N_ cells and expansion of T_EM_ cells (and CD8^+^ T_EMRA_) within the lymphocyte population (Fig. 5a). Unlike the CD4^+^ T lymphopenia and increased cytokine production seen in RRMM, these changes were also already present in NDMM, in particular within the CD8^+^ T cell population. T cell subsets were broadly comparable between PBMC and BMMC samples from NDMM patients, except that T_EMRA_ made up a larger proportion and T_CM_ a smaller proportion of BM as compared with PB CD4^+^ T lymphocytes (Fig. 5b).

Mirroring the human findings, in transgenic Vk*MYC BMMC, there was a reduction in the proportion of T_N_ (and T_CN_ in the mouse model) with an increase in T_EM_ within the CD4^+^ and CD8^+^ T lymphocyte populations with disease progression (Fig. 5c). The expansion of T_EM_ within the CD8^+^ T cell population was most apparent (Fig. 5c(iii)) and is likely to account for increased IFNγ expression from CD8^+^ T cells; indeed there was a correlation between the two (Fig. 5d). Similar changes in the CD8^+^, but not CD4^+^, T memory cell profile were noted in a diseased cohort of transplant Vk#4929 (Fig. 5e).

### Markers of T cell exhaustion are altered in human MM patients, even at first diagnosis

**Fig. 6:**
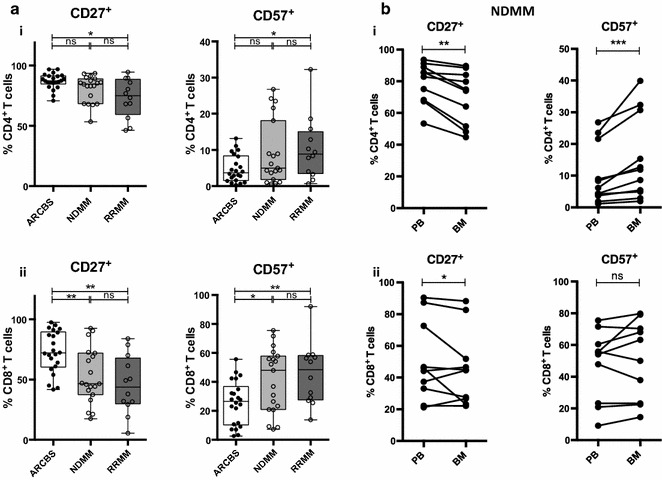
CD27/CD57 expression analysis in human MM. **a** Human PBMC CD4^+^/CD8^+^ T cell analysis of CD27 and CD57 expression. Box and whisker plots showing % CD27^+^ (*left*) and  % CD57^+^ (*right*) of (*i*) CD4^+^ T cells and (*ii*) CD8^+^ T cells. *Clear boxes* ARCBS donors, *light grey boxes* NDMM, *dark grey boxes* RRMM. **b** CD27 and CD57 expression matched pair analysis in PBMC and BMMC from NDMM patients (as described in **a**). (*i*) CD4^+^ T cells (*ii*) CD8^+^ T cells

We additionally analysed levels of the co-stimulatory molecule CD27 and the senescence-associated marker CD57, which have been shown to be altered in CD8^+^ T lymphocyte populations in elderly individuals [10, 29, 30] and in MM [32, 33]. In NDMM and, most notably, RRMM patients, CD8^+^ T cells exhibited reduced CD27 expression and increased CD57 in keeping with an increasing proportion of antigen experienced T cells. These changes were also seen in CD4^+^ T cells in RRMM (Fig. 6a). BM CD4^+^ T cells demonstrated lower CD27 and higher CD57 levels than PB CD4^+^ T cells (Fig. 6b(i)) that is likely explained by the larger proportion of T_EMRA_ in BM (Fig. 5b(i)). BM CD8^+^ T cells exhibited slightly lower CD27 levels but equivalent levels of CD57.

## Discussion

We set out to compare T cell profiles in NDMM and RRMM, and to establish whether the immunology is replicated in the Vk*MYC mouse models. Our analysis of patient samples illustrates that the lymphocyte profile is already changing at the time of diagnosis of MM, with the development of B lymphopenia [5, 6] and a shift in the T cell profile from a predominantly naïve to a predominantly antigen-experienced T_EM_/T_EMRA_ cell population (Fig. 5), associated with markers of T cell exhaustion (Fig. 6). These aberrancies are even more apparent with progression to RRMM; however, significant additional immunological changes have occurred that include CD4^+^ T lymphopenia [8] and increased inflammatory cytokine production (Fig. 4).

This established a baseline against which the immunology of mouse models could be compared; though we acknowledge that, because of pragmatic limitations with the availability of both PB and BM human MM samples, direct comparison with murine BM may not fully address compartmental differences. Nevertheless, alterations in BM inflammatory T cell profiles have been mirrored in PB [28, 34], and we have shown broadly similar T cell profiles in PB and BM in NDMM samples (Figs. 4b, 5b).

In our analysis of the mouse models and other published data [19, 35] we found that, regardless of whether it is the transgenic or transplant version of Vk*MYC, the amount of BM disease appears to be a critical factor in determining changes to cellular immunity. In all Vk*MYC mouse models, increasing tumour burden was associated with a progressive B lymphopenia (Fig. 2b, c); however, alterations in the T cell profile differed. High-grade disease in transplant Vk#31 was clearly associated with an inflammatory CD8^+^ T cell profile, reminiscent of RRMM. This might have been replicated in the transgenic Vk*MYC mice had there been a higher burden of disease: Calcinotto et al. [19] described BM accrual of CD4^+^ and CD8^+^ T lymphocytes and an inflammatory T cell profile in the transgenic Vk*MYC model, which could be explained by the higher BM plasma cell infiltrates (i.e. 40–80 %) in their cohort as compared to ours (i.e. 30–50 %). The lack of any inflammatory T cell response in transplant Vk#4929 might be explained by the fact that it is a very aggressive clone that causes rampant extramedullary disease and may not allow time for the usual immune aberrancies to develop. In line with this observation, another group described CD8^+^ T lymphopenia developing in mice transplanted with an aggressive Vk*MYC clone (mice needed to be culled by 5 weeks post-transplant) [35]. There is also the suggestion that inflammation wanes with end-stage disease [35] or is replaced by a Th2 response [19].

The Vk*MYC model most successfully replicates changes within the memory T cell population, i.e. from a predominantly naïve to predominantly effector T cell population, observed in patients with MM (Fig. 5) and with advancing age [10]. This is noteworthy in the tumour setting, as effector memory CD8^+^ T cells have been shown to exhibit inferior anti-tumour immunity to central memory CD8^+^ T cells [36], despite being efficient IFNγ producers [37]. Similar findings were recently reported in another mouse model of chronic haematological malignancy [38]: in the Eμ-TCL1 mouse model of chronic lymphocytic leukaemia (CLL), the authors described the expansion of IFNγ-producing CD8^+^ T cells in ageing transgenic mice, and effector memory T cells (at the expense of naïve T cells) in normal and transgenic ageing mice, as well as mice adoptively transplanted with CLL. They also reported aberrant PD1 expression on CD44^+^CD3^+^CD8^+^ T cells, suggesting an exhausted phenotype.

Appreciating T cell populations and their fate with progressive disease is essential to understanding immune evasion by malignancies, but is also very relevant in mouse models that are used in a translational capacity to develop novel therapies. It is likely that immunotherapeutic treatments, which rely on the presence of a T and NK cell response, have therapeutic “windows” throughout the natural progression of MM, i.e. some treatments might be better utilized earlier in the course of disease before T cell immunosenescence has occurred.

We suggest that there may be a “window” akin to NDMM for testing immunomodulatory drug therapies in the transgenic Vk*MYC model, but the transplant Vk#31 model and advanced disease in the transgenic Vk*MYC model more accurately replicate the T cell immune microenvironment of RRMM (or even end-stage disease in transplant Vk#4929). The fact that the Vk*MYC mouse model most closely correlates with RRMM is perhaps not so surprising, given that it relies on the overexpression of the *MYC* oncogene that is more commonly associated with advanced human MM. Of notable interest, it was recently demonstrated in a mouse model of *MYC*-induced T cell acute lymphoblastic leukaemia, that *MYC* could regulate the expression of two immune checkpoint inhibitors, CD47 and PD-L1, on the tumour cell surface and thereby suppress CD4^+^ T cell, CD69^+^ activated T cell and F4/80^+^ macrophage recruitment [39].

There is clearly much more to be learnt about the immunology of *MYC*-driven malignancies, beyond its role in tumourigenesis. However, in the Vk*MYC mouse models, we would suggest that the most appropriate role for testing immunomodulatory therapies is likely to be in RRMM that remains an area of clinical need for novel therapies.

## Conclusions

Our findings indicate that T cell profiles differ in patients with newly diagnosed MM from that seen in advanced MM. Advanced disease in transgenic and transplanted Vk*MYC mouse models of MM more closely mirrors the immunology of patients with RRMM rather than NDMM. This is an important consideration when interpreting the pre-clinical findings of therapies tested in the Vk*MYC mouse model.

## Abbreviations

a-m WT: age-matched wild type
ARCBS: Australian Red Cross Blood Service
BM: bone marrow
BMMC: bone marrow mononuclear cells
CBA: cytometric bead array
CLL: chronic lymphocytic leukaemia
FS/SS: forward scatter/side scatter
ICS: intracellular cytokine staining
MGUS: monoclonal gammopathy of undetermined significance
MM: multiple myeloma
NDMM: newly diagnosed multiple myeloma
Ns: not significant
PB: peripheral blood
PBMC: peripheral blood mononuclear cells
RRMM: relapsed/refractory multiple myeloma
T_CM_: central memory T cell
T_EM_: effector memory T cell
T_EMRA_: terminally differentiated effector memory T cell
T_N_: naïve T cell
TG: transgenic
TP: transplant
